# Activation of persons living with HIV for treatment, the great study

**DOI:** 10.1186/s12889-015-2382-1

**Published:** 2015-10-16

**Authors:** Kevin Fiscella, Michele Boyd, Julian Brown, Jennifer Carroll, Andrea Cassells, Roberto Corales, Wendi Cross, Nayef El’Daher, Subrina Farah, Steven Fine, Richard Fowler, Ashley Hann, Amneris Luque, Jennifer Rodriquez, Mechelle Sanders, Jonathan Tobin

**Affiliations:** Public Health Sciences and Community Health, Department of Family Medicine, University of Rochester Medical Center, 1381 South Ave, Rochester, NY 14620 USA; Public Health Sciences, University of Rochester Medical Center, Rochester, USA; Trillium Health, Rochester, USA; Department of Family Medicine, University of Colorado, Denver, USA; Clinical Directors Network, New York, USA; Department of Psychiatry, University of Rochester Medical Center, Rochester, USA; Rochester Regional Health System, Unity Family Medicine at St. Mary’s and Unity Infectious Disease, Rochester, USA; Department of Medicine, Infectious Diseases (SMD), University of Rochester, Rochester, USA

**Keywords:** Patient activation, HIV, Health information technology

## Abstract

**Background:**

Patient empowerment represents a potent tool for addressing racial, ethnic and socioeconomic disparities in health care, particularly for chronic conditions such as HIV infection that require active patient engagement. This multimodal intervention, developed in concert with HIV patients and clinicians, aims to provide HIV patients with the knowledge, skills, attitudes and tools to become more activated patients.

**Methods/Design:**

Randomized controlled trial of a multimodal intervention designed to activate persons living with HIV. The intervention includes four components: 1) use of a web-enabled hand-held device (Apple iPod Touch) loaded with a Personal Health Record (ePHR) customized for HIV patients; 2) six 90-minute group-based training sessions in use of the device, internet and the ePHR; 3) a pre-visit coaching session; and 4) clinician education regarding how they can support activated patients. Outcome measures include pre- post changes in patient activation measure score (primary outcome), eHealth literacy, patient involvement in decision-making and care, medication adherence, preventive care, and HIV Viral Load.

**Discussion:**

We hypothesize that participants receiving the intervention will show greater improvement in empowerment and the intervention will reduce disparities in study outcomes. Disparities in these measures will be smaller than those in the usual care group. Findings have implications for activating persons living with HIV and for other marginalized groups living with chronic illness.

**Trial registration:**

ClinicalTrials.gov Identifier: NCT02165735, 6/13/2014.

## Background

There are large, potentially avoidable differences in the incidence, prevalence and outcomes of HIV infection [1]. Combination antiretroviral therapy (cART) represents a powerful treatment with the potential to dramatically reduce disparities in morbidity and mortality from HIV. cART has radically altered the course of HIV and transformed it into a chronic disease with a life expectancy similar to adult onset diabetes [2]. Successful management of HIV-infection requires patients to be empowered, (i.e. engaged, informed, collaborative, committed, and tolerant of uncertainty). HIV disease control requires highly individualized cART based on consideration of multiple factors. These include viral genotype, short and long-term side effects and toxicity, dosing frequency, pill burden, potential drug-drug interactions, comorbidities, and most importantly, consideration of patient preferences and values [3]. Viral suppression, (i.e. attainment of undetectable viral loads), requires long-term rates of medication adherence that exceed 95 % [4]. Effective clinical management requires near complete suppression of viral replication in order to restore immune function, minimize viral mutations and drug resistance, prevent opportunistic infections, and decrease risk of transmission of HIV infection to others [4].

Poor and minority patients are less likely to be active partners in their own care [5]. Poor and minority patients, including persons living with HIV (PLWH), report lower partnership levels with their clinicians, [6] miss more office visits, [7] ask fewer questions during their visits, [8–10] report less confidence in self-management,[11] and more frequently miss doses [12] or stop taking their cART [13]. Groups that have been historically disempowered also have lower knowledge of their medications, [14] are less likely to report benefits from cancer screening, [14] and less likely to report involvement in cancer screening decisions [15].

Our intervention, developed and piloted in collaboration with PLWH, clinicians in Federally Qualified Health Centers (FQHCs); Patient-Centered Medical Home and a primary care practice-based research network (PBRN), will address these disparities by focusing on empowerment and information access that are needed to make decisions that reflect patients’ values.

## Study aims and hypotheses

The overarching aim of this project is to assess the impact of a multi-model eHealth group behavioral intervention on patient activation among persons living with HIV. The specific aims and hypotheses are shown in Table 1 below.

**Table 1:** Study Aims and Hypotheses

Aim 1: Improve PLWH’s empowerment
H 1.1: We will improve patient activation; decision making ability; and perceived knowledge, comfort, and perceived skills at finding, evaluating, and applying electronic health information to health problems.
H 1.2: We will improve clinicians’ communication skills as perceived by PLWH.
Aim 2: Increase PLWH’s receipt of evidence-based care
H 2.1: We will improve patient confidence in adhering to combination antiretroviral treatment (cART), self-reported adherence to cART, and HIV viral suppression (undetectable viral load).
H 2.2: We will increase receipt of evidence-based clinical preventive services.
Aim 3: Improve PLWH’s health
H 3.1: We will improve mental, social, and overall health
Aim 4: Reduce disparities in PLWH’s empowerment
H 4.1: We will produce the greatest improvement in activation for those with lowest baseline activation.
H 4.2: We will observe comparable improvements by race, ethnicity, and education.

## Methods

### Study design

We will conduct a parallel randomized controlled trial. This design allows us to examine pre- and post-assessments of participants randomized to the intervention (treatment group) compared to participants randomized to usual care (control group). Given our focus on individual patients, the nature of our intervention, including its reliance on training and specific tools, the risk for contamination between participants will be relatively small. Any such contamination will bias results towards the null. We considered other designs including randomizing by practice site (cluster RCT), stepped wedge and staggered enrollment trials, but did not choose these designs due to few clusters, data collection burden, and relatively short funding time frame (3 years).

### Eligibility

To maximize generalizability, we opted for a pragmatic trial that employs broad inclusion criteria of participants.

#### Inclusion criteria

>18 years, confirmed HIV diagnosis, and receiving care within a participating site.

#### Exclusion criteria

Inability to provide informed consent, inability to read and speak English.

### Participant recruitment and enrollment

Patients will be recruited from four participating sites in Rochester NY and from four sites in the New York Metropolitan Area. Most of the Rochester sites are dedicated HIV practices that also provide primary care to some of their patients, while all of the NYC sites are Federally Qualified Health Centers (FHQC) that provide primary care, including HIV care. Clinicians and staff in participating sites will refer interested HIV+ patients to a study research assistant (RA). The RA will meet in a private area and review the study protocol and consent patients.

### Procedures for randomization and allocation

Sequential identification (ID) numbers will be generated by our study biostatistician using computer generated random numbers stratified by site (Rochester or NYC) to minimize potential site variation between groups. Assignments will be concealed in sealed opaque, envelopes that contain the group assignment. Following confirmation of the participant’s eligibility, informed consent, and completion of the baseline assessment (T0), the RA will open the envelope and notify the participant whether they have been assigned to the intervention or usual care group.

### Procedures and timing of data collection

The RA will administer surveys to all participants at three times: T0 (baseline); T1 (8–10 weeks following randomization), and T2, (1–3 days following the patients’ next HIV visit which is using 14–24 weeks after baseline). We have chosen to link the timing of the T2 survey to patients’ HIV visits in order to ensure reliable patient recall of the visit. Most patients have HIV visits every 3–4 months, and their HIV Viral Load is routinely measured at these visits. T3 (>52 weeks following randomization) involves chart abstraction. RAs or participating clinical site staff trained in chart abstraction, who are blinded to treatment allocation, will abstract the data from the EHR. Fidelity checks will be conducted on 10 % of the sample by an RA and/or study coordinator in order to achieve >90 % agreement.

### Data management and quality control

An RA will enter data into a laptop computer and upload it into the Web-based RedCap database [16]. Invalid responses and outliers (those falling outside of response ranges) will be corrected immediately. Quality reports will be conducted weekly using RedCap analytic tools to assess missing data and rates of invalid responses. All laptops will be fully encrypted and password protected. Files linking participant names with study ID numbers will be stored separately on a secure, password protected server.

### Group facilitator training and fidelity

We have conducted group training of group facilitators for the intervention from Rochester and NYC using combined in-person and webcasts/video tele-conferences. The training focused on key knowledge, skill, and attitudes (KSAs) for trainees to facilitate groups and also provide individual coaching. These KSAs include knowledge of curriculum content including use of the device and ePHR, skills in facilitating adult learning in groups, and enthusiasm and expectation that every learner will succeed. The facilitator training was based on application of the steps specified in the training manual. We used direct observation and video recording to review key skills and practice until the specified behavior could be performed with high fidelity. During the intervention phase, the facilitators will also meet monthly and use fidelity rating check lists to ensure adherence. A national expert in fidelity rating will assist with fidelity training, refinement of the fidelity checklist, and analyses of protocol adherence during training sessions.

### Intervention

The intervention primarily focuses on patient activation. It includes group sessions to teach participants how to use the hand-held device, how to use the electronic patient health record (ePHR), and how to effectively communicate with the patients’ HIV clinician.

#### Group training

Group training, co-learning and sharing is critical to facilitating patient empowerment. While an iPod represents “a hook” to encourage participants to enroll, we explicitly target key *intrinsic motivational factors* (autonomy, competence, and relatedness) as a means for energizing motivation to learn to use and apply empowerment tools and skills, and to encourage attendance, participation (dose) and retention (maintenance) in the intervention. Each of the six 90-min sessions focuses on development of a basic competency within a context that supports patient autonomy, competence and human relationships. Content is integrated (i.e. role playing is mixed with app downloading) to maintain enthusiasm. A training manual appears in the Appendix. Key tasks are summarized below: (Table 2).

**Table 2:** Key tasks for group training

•Project overview, value affirmation exercise, and basic use of the device (e.g. turning on and off, password, settings, etc.)
•Basic training in use of ePHR (recap and introduction to features, password, back-up, and importing contacts).
•Advanced training in use of ePHR (reminders, data entry, confirmation of data, To Ask list).
•Communication and use of web sites (training in formulating, prioritizing, asking questions, and bookmarking high quality web sites).
•Communication and health apps (role play challenging situations, using ePHR during visit, and downloading health relevant apps).

#### Hand-held device

For the hand-held device, we selected the Apple iPod Touch® based on its widespread availability and adoption, versatility (number of applications, web-enabled), intuitive interface/usability, relative low cost, no requirement for phone or internet service provider contracts, and its “coolness” factor. The latter is important to promoting initial patient engagement, including new membership *in the technology community*. This helps improve patients’ inclusion in mainstream society which has greater levels of web-access (both cellular and ISP-based).

#### ePHR

We will load each device with our customized ePHR developed for PLWH named *URHealth. URHealth* possesses the following features. 1) Drop down menus for common HIV medications with accompanying pill pictures; 2) Common lab tests with brief, understandable explanations. 3) Ability to set reminders for appointments and medications. 4) Personalized “Prompt list” of potential questions for patient to ask their clinician. These are generated based on patient’s age, date of birth and data the patients enter into their device. For example, if the person is under 26 years old, they will be prompted to ask about the HPV vaccine (unless they have entered dates of receipt). Prompts can be personalized by prioritizing them. They can be augmented using personally relevant questions that participants will be trained to generate for themselves. The app is password-protected, adding an additional layer of security over the iPod itself.

#### Pre-visit coaching

After participants in the intervention group have completed their last session, the RA will meet with the patient prior to their next office visit with their HIV provider. This one-time individual coaching session is designed to reinforce application of skills learned during the group training. A separate manual and fidelity checklist has been developed for this one-on-one session. Key tasks for the session are summarized below (Table 3).

**Table 3:** Key tasks for pre-visit coaching

•Bring iPod/ePHR (reminding the patients to bring their iPod to their patient coaching session and to their subsequent doctor visit).
•Preparation of questions (ensuring that the patient has identified and recorded in their iPod key questions to ask their clinician).
•Application and rehearsal of skills (coaching the patient through role play asking his/her questions.

#### Clinician training

We will train clinicians to engage patients in the intervention group, (i.e. those who present with their ePHR in the visits) through an educational “lunch and learn” session that is CME-accredited. These educational sessions will address the purpose of the project and basic clinician behaviors to facilitate empowerment. Drs. Carroll and Fiscella will lead these sessions. We will use training approaches used in previous studies [17–19] that are supported by systematic reviews of randomized controlled trials of clinician training [20, 21]. We include explicit demonstrations using brief videos [22]. We will show examples of effective and ineffective communication. These include encouraging the patient to use their ePHR for questions, praising patients for engagement, helping patients prioritize their questions, and providing patients with relevant information to enter into their ePHR. Clinicians will be encouraged to download URHealth onto their own personal devices, in order to familiarize themselves with the ePHR and to enable them to provide ongoing support and effective answers to questions from their patients about use of the ePHR.

### Training manual

We have developed a detailed training manual for instruction of project staff and peers as facilitators to conduct the patient group sessions. The onsite train-the-trainers sessions will focus on competency in conducting each of the six group sessions with specific attention to displaying enthusiasm, active engagement of group through questions and sharing, staying on task and time, and ensuring all members are able to rehearse particular skill during the session. We will assess fidelity using an observation checklist applied to intervention group videotapes. All train-the-trainer sessions will be recorded to train new staff as well as provide refresher training if drift occurs. After study completion, these trainings will be posted online to enhance dissemination and sustainability.

### Usual care

Participants assigned to this group will receive usual care from their HIV clinician during the duration of the study. They will receive the same surveys at the same time periods, T01–T3 as those assigned to the intervention group. Following the completion of their final assessment (T3), participants in the usual care group will receive the iPod loaded with the URHealth application, and will be provided with the training manual to assist with self-teaching on the use of the application.

### Study outcomes and measures

Our study measures reflect our patients’ choice of outcomes related to empowerment including activation, eHealth literacy, and greater decision-making and involvement in care. These outcomes will be assessed using validated scales with sound psychometric properties and through chart abstraction data. These are summarized below (Table 4).

**Table 4:** Study measures, data source, and timing

Construct (Hypothesis)	Measure	Data type source	Collection points
Participant characteristics			
Demographics	Standardized questions	Survey	T0
Computer experience	Standardized questions	Survey	T0
Health history	Standardized questions	Survey	T0
Aim 1 (Empowerment)			
Patient activation (H1.1)	PAM [56, 57]	Scale	T0, T1, T2
Decision making (H1.1)	Decision Self Efficacy Scale [26]	Scale	T0, T1, T2
eHealth Literacy (H1.1)	eHEALS [23, 24]	Scale	T0, T1, T2
Involvement in Care (H1.2)	Perceived Involvement in Care [25]	Scale	T0, T1, T2
Aim 2 (Evidence-based care)			
HIV adherence self-efficacy (H2.1)	ASES [29]	Survey	T0, T1, T2
Adherence to cART (H2.1)	Past week adherence [28]	Survey	T0, T1, T2
HIV viral load <50 (H2.2)	HIV viral loads (both actual level; and whether undetectable or not)	Chart abstraction	T0, T2
Evidenced based preventive care	Cancer screening/immunizations [58]	Chart abstraction	T0, T3
Aim 3 (Health)			
Physical and Mental health/Quality of Life (H3.1)	SF-12 [27]	Scale	T0, T1, T2
Aim 4 (Moderators)			
Low Activation (H4.1)	PAM and eHEALS (<median)	Scales	T0
Minority race/ethnicity (H4.2)	Standardized questions	Survey	T0
Low education (4.2)	Standardized questions	Survey	T0
Clinician patient centeredness	Instrument on Doctor-Patient Communication Skills. [30]	Scale	

#### Patient empowerment

Our primary outcome is patient empowerment assessed using the Patient Activation Measure (PAM). We will capture related additional dimensions of empowerment including eHealth literacy (eHealth Literacy Scale eHEALS [23, 24]), patient involvement in their care (Patient Involvement in Care [25]) and, patient confidence in their health care decision making (Decision Making Self Efficacy Scale [26]).

#### Patient health status

We assess patient’s mental, social and overall health based on the well-validated Medical Outcomes Study SF–12 [27]. It provides an overall score and relevant subdomain scores.

#### Adherence measures

We will assess adherence and receipt of evidence-based care using a series of adherence related measures and data abstracted from the EHR. These include validated measures of adherence to cART [28] and patient self-efficacy related to adherence, [29] in addition to viral load values (from medical records) and a summary index of evidenced-based care (from medical records). The index will be derived based on receipt of the number of evidence-based measures received divided by the total number the patient is eligible for. These include those receiving an A or B rating by the US Preventive Services Task Force, e.g. breast, cervical and colorectal cancer screening, immunizations, lipid screening,

#### Patient social disadvantages

We will assess race (White, Black, Asian, Other) and ethnicity (Latino or not), age, and sex based on self-report. We define low education as absence of any high school degree. Low activation and low eHealth literacy will be based on less than the median value on the PAM and eHEALS.

#### Clinician communication

We will assess the communication skills of clinicians using the Doctor-Patient Communication Skills instrument [30]. This validated scale assesses key aspects of clinician communication from the perspective of the patient. We will minimize patient reporting bias by aggregating clinician scores across patients. We chose to use patient ratings of clinicians rather than observer ratings because these are more patient-centered measures and patients’ perceptions probably have the greatest impact on patients’ behavior; as well as their lower costs and greater capacity for pragmatic implementation. We chose this scale over other patient reported measures of clinician communication because it comes closest to measuring the clinician competencies that we address in our clinician training and which our patients tell us are most meaningful to them.

### Preliminary data

We piloted the intervention with 32 PLWH.

We obtained pre and post assessments on the participants and assessed change scores. Our pilot data below (Table 5) show improvement in key measures. In addition, participants with the least education, activation, and least eHealth literacy benefited the most. Specifically, those with less than a high school education and those with lower activation or lower eHealth literacy (i.e. below the median for the group) showed larger improvements in adherence, eHealth literacy, perceived involvement in care, decision in self-efficacy, and patient activation than those with higher levels.

**Table 5:** Characteristics of PLWH in pilot study (*n* = 32)

Age mean (Standard deviation)	49 (10.2)
Gender^a^	
Female	16 (50 %)
Male	15 (47 %)
Race/ethnicity	
Hispanic	8 (25 %)
Non-Hispanic White	1 (3 %)
Non-Hispanic Black	20 (63 %)
Other	3 (6 %)
Computer Use (% none)	7 (22 %)
Insurance Type	
Public (Medicaid/Medicare)	30 (94 %)
Private	0 (0 %)
ADAP	4 (6 %)
Education	
<High school	10 (31 %)
High school diploma or GED	6 (19 %)
More than high school	16 (50 %)
Annual Income^a^	
<$20,000	29 (91 %)
$20,000–29,999	2 (6 %)
≥$30,000	0 (0 %)

^a^Note not all categories total 100 % due to missing values

### Planned analysis

Following collection and cleaning of data (T0–T3), the underlying assumptions of all analyses will be checked using appropriate graphical and numerical methods [31]. Data accuracy will be investigated upon discovery of any outliers or influential data points. If an outlier is detected, we will truncate the outlier by assigning a reasonable value or replacing it using the adjacent values from the remaining data. We will try to utilize all data points in the final analysis.

The proposed study adopts a randomized repeated measures pre-test/post-test design and our analytic plan reflects this design. We will use generalized estimating equations (GEE) to obtain robust estimates and to account for the repeated measures study design and the correlated data structure of subjects nested within study site [32]. The chosen link function for the models will be appropriate for the distribution of each outcome. Unless otherwise stated, all statistical tests will be performed at the two-tailed 5 % level of significance. Likewise, 95 % confidence intervals will be constructed for estimation of effects (e.g., difference in outcomes between intervention and control groups). We will use Dunnett and Hsu’s methods to control for multiple comparisons in secondary outcomes [33]. All analyses will be performed with SAS® statistical software version 9.3 (SAS Institute, Cary NC).

We will summarize frequencies of categorical variables and means or medians for continuous and ordinal variables. Continuous variables with skewed distributions will be transformed to approximate normality. We will construct tables that display baseline characteristics for participants, overall and by (blinded) group assignment. We will compare baseline information on participants including demographic characteristics, previous computer use, and health factors. We will assess whether attrition differs across baseline characteristics or by group assignment and adjust for differences if present using multiple imputation if necessary. We will conduct intention-to-treat analyses.

Every effort will be made to facilitate and encourage participants’ completion of all surveys. In the event that missing data occur, we will attempt to contact participants and obtain the data or to find out why the surveys or items are missing. The reasons for missing data will be documented and the mechanism for missing data will be examined [34]. If needed, data will be imputed and a weighted generalized estimating equations (WGEE) model will be applied using the inverse probability of missingness as the sample weight [35]. Sensitivity analyses will be performed to determine the impact of key assumptions.

### Aim 1: Improve PLWH’s empowerment

To assess H 1.1 (Improvement in patient activation; decision making ability; and perceived knowledge, comfort, and perceived skills at finding, evaluating, and applying electronic health information to health problems), we will compare changes in patient activation (primary outcome), eHealth literacy, and decisional self-efficacy outcomes by assigned randomization group using GEE models that control for baseline scores. All models will be adjusted for any potential confounders identified by the initial analyses. We will use the same approach to assess H 1.2 (substituting Perceived Involvement in Care).

### Aim 2: Increase PLWH’s receipt of evidence-based care

To assess H 2.1 (improvement in patient improve patient confidence in adhering to combination antiretroviral treatment (cART), self-reported adherence to cART, and HIV viral suppression), we will substitute these dependent variables in the above models. Similarly for H 2.2 (improvement in evidence-based care), we will index for evidence-based care for the dependent variable. This index will be created by passed on ratio of interventions that the patient received versus the number they were eligible for.

### Aim 3: Improve PLWH’s health

To assess H 3.1 (improvement in mental, social and overall health) we will use the same approach substituting the scores from the SF-12 physical and mental health components.

### Aim 4: Reduce disparities in PLWH’s empowerment

To assess H 4.1 (greatest improvement in patient activation will be seen in those with lowest baseline activation scores), we will enter interaction terms into the models with treatment assignment. The interaction terms will be based on median cut-offs for activation. To assess H 4.2. (comparable improvements in by race, ethnicity and education). We repeat the above process using an interaction term between treatment assignment and the sociodemographic characteristics.

### Power

The number of participants included in the intervention group and the usual care groups is based upon a series of power analyses. For H 1.1 – H 3.1, we estimated detectable difference using the method described by Donner and Klar, [36] using 8 study sites total, a significance level of 0.05, and a minimum power of 80 %. The intra-cluster correlation (ICC = 0.0207) was estimated from our pilot data and is consistent with other studies [37]. We plan to enroll 360 participants to allow for attrition and assume a 15 % drop-out rate. Thus, we will be able to detect an effect size of 0.51 for each of the measures in Table 6. For H 4.1 and H 4.2, we ran Monte Carlo simulations to determine that we will have a minimum of 80 % power for the interactions [38].

**Table 6:** Change in empowerment dimensions (effect size) by participant education, activation or eHealth literacy

Empowerment dimension	<High school	High school	>High school	Low activation	High activation	Low eHealth literacy	High eHealth literacy
HIV adherence self-efficacy	0.15	−0.64	−0.19	0.18	−0.38	−0.02	−0.05
eHealth literacy	0.85	0.35	0.61	0.98	0.18	0.94	0.35
Perceived Involvement in Care	0.57	0.37	0.24	0.61	0.11	0.48	0.26
Decision self-efficacy	0.81	0.23	0.34	0.86	0.16	0.48	0.52
Patient Activation	0.50	−0.85	0.24	0.52	−0.155	0.32	0.11

### Funding support, trial registration, ethics approval

This trial is exclusively funded by PCORI. The trial is registered at ClinicalTrials.gov (NCT02165735). Ethical approval was obtained from the Institutional Review Boards of University of Rochester and Clinical Directors Network (CDN) and Lutheran Family Health Center Network IRB.

## Discussion

Poor and minority patients including PLWH report lower engagement in their care, [6] miss more office visits, [7] ask fewer questions during their visits, [8–10] report less confidence in self-management, [11] and more frequently miss doses [12] or stop taking their cART [13]. Groups that have been historically disempowered also have lower knowledge of their medications. [14] These disparities likely contribute to disparities in adherence, [39, 40] viral suppression, [41, 42] and ultimately - to disparities in HIV treatment outcomes, [43–45] including hospitalizations and mortality [2, 46].

These disparities in engagement may be further exacerbated by the well-documented digital divide. Federal meaningful use standards are driving adoption of online PHRs for patients [47]. Lower use of these online portals by lower income and minority patients, [48–55] could further exacerbate current disparities in patient engagement by creating digital barriers to access to test results, requests for refills and appointments, and electronic messaging with clinicians.

This intervention is among the first to explicitly target these disparities among PLWH using a unique training program that focuses on self-management using a hand held PHR. Unique features include an explicit focus on use of peer-trainers, participant-to-participant teaching and sharing about HIV self-management and health technology, use of a customized PHR for PLWH, and explicit training in communication with clinicians linked to use of the PHR.

Perhaps most notably, the design of the intervention and study emerged using a community-based participatory research model involving collaboration between PLWH, clinicians and HIV organizations. This active partnership has ensured that all aspects of the intervention are tailored to the needs of PLWH, including those with low eHealth literacy and that measures capture key outcomes meaningful to patients, particularly empowerment, which we have operationalized using the patient activation measure. The study design helps ensure rigorous evaluation of the intervention including benefit to those with lower levels of activation and eHealth literacy.

The primary limitation is that it will not be possible to determine for certain which element of this multimodal intervention improves outcomes the most. However, proposed mediational analyses may shed some light on particular salient pathways. If shown to be effective, this study will provide an intervention that can be generalized to support self-management for other chronic diseases, and which can be disseminated, scaled up and sustained over time.
